# Factors influencing mortality in abdominal solid organ transplant recipients with multidrug-resistant gram-negative bacteremia

**DOI:** 10.1186/s12879-017-2276-1

**Published:** 2017-02-27

**Authors:** Bingbing Qiao, Jianzhen Wu, Qiquan Wan, Sheng zhang, Qifa Ye

**Affiliations:** 1 Department of Hepatobiliary and Pancreatic Surgery, the First Affiliated Hospital of Zhengzhou University, Zhengzhou 450052, Henan, People’s Republic of China; 2 Department of Cadre Care, the Third Xiangya Hospital, Central South University, Changsha 410013, Hunan, People’s Republic of China; 3 Department of Transplant Surgery, the Third Xiangya Hospital, Central South University, Changsha 410013, Hunan, People’s Republic of China; 4 Department of Transplant Surgery, Zhongnan Hospital, Wuhan University, Wuhan 430071, Hubei, People’s Republic of China

**Keywords:** Multidrug-resistant, Gram-negative bacteremia, Predictors, Mortality, Abdominal solid organ transplantation

## Abstract

**Background:**

Although multidrug-resistant (MDR) gram-negative bacteremia (GNB) has been recognized as an important cause of morbidity and mortality among abdominal solid organ transplant (ASOT) recipients, there are no data on its prognostic factors after an interim standard definition of MDR was proposed in 2012. The objective of this study was to describe the epidemiology, microbiology, and predictors of infection-related 30-day mortality in ASOT recipients with MDR GNB.

**Methods:**

We performed a retrospective, double-center analysis of ASOT patients with MDR GNB over a 13-year study period. Univariate and multivariate analyses were performed to identify the risk factors for mortality.

**Results:**

During the observational period, 2169 subjects underwent ASOT. Ninety-nine episodes of MDR GNB were diagnosed in 91 (4.6%) ASOT recipients, with a predominance of *E.coli* (29 isolates, 29.3%) and *A.baumanii* (24 isolates, 24.2%). The median age of these 91 recipients was 45 years (interquartile range 35–54). Mortality after the first episode of MDR GNB was 39.6% (36 deaths). The univariate analysis identified the following variables as predictors of MDR GNB-related mortality: lung focus (*P* = 0.001),nosocomial origin (*P* = 0.002), graft from donation after cardiac death or deceased donors (*P* = 0.023), presence of other concomitant bloodstream infection (*P* < 0.001), temperature of 40 °C or greater at the onset of MDR GNB (*P* = 0.039), creatinine > 1.5 mg/dl (*P* = 0.006), albumin < 30 g/L (*P* = 0.009), platelet count < 50,000/mm^3^ (*P* < 0.001), and septic shock (*P* < 0.001). In the multivariate logistic regression analysis, septic shock (odds ratio (OR) = 160.463, 95% confidence interval (CI) = 19.377–1328.832, *P* < .001), as well as creatinine > 1.5 mg/dl (OR = 24.498, 95% CI = 3.449–173.998, *P* = 0.001), nosocomial origin (OR = 23.963, 95% CI = 1.285–46.991, *P* = 0.033), and presence of other concomitant bloodstream infections (OR = 27.074, 95% CI = 3.937–186.210, *P* = 0.001) were the variables associated with MDR GNB-related 30-day mortality.

**Conclusions:**

MDR GNB was associated with high morbidity and mortality in ASOT recipients, with a predominant causative organisms being *E.coli* and *A.baumanii*. Nosocomial origin, as well as presence of other concomitant bloodstream infections, increased creatinine level and septic shock were the main predictors of MDR GNB-related 30-day mortality.

## Background

Bloodstream infection (BSI) frequently complicates the postoperative course and is the most frequent life-threatening infectious complication after abdominal solid organ transplantation (ASOT) [1–10]. The morbidity and mortality rates have been reported to range from 4.5-69% [2, 11–22] and 2.4–52% [2, 5, 6, 15, 16, 21, 23–30] in ASOT recipients with BSI, respectively.

Significantly, recent reports have demonstrated a shift in the distribution of BSI toward Gram-negative bacteria as the predominant pathogens and multidrug-resistant (MDR) gram-negative bacteremia (GNB) have emerged frequently after transplantation [20, 31–33]. Among liver recipients with BSI, 67% of Enterobacteria and 75% of non-fermenters were MDR [31]. The mortality rate was 44.4 and 36.6% in MDR non-fermenters and *P.aeruginosa* bacteremic transplant recipients, respectively [2, 34]. Aguiar EB et al. found that 26% of ASOT patients died within 30 days of the diagnosis of the first episode of bacteremia caused by extended-spectrum β-lactamase (ESBL)-producing Enterobacteriaceae [35]. BSI or non-BSI caused by carbapenem-resistant *K.pneumoniae* led to a staggering up to 82% mortality after liver transplantation [36–40]. The mortality due to extensively drug-resistant *A.baumannii* infections, with 88% of these infections being ventilator-associated pneumonia, was 56% among solid organ transplant (SOT) recipients [41].

Although the emergence of MDR GNB in SOT recipients has been demonstrated in multiple studies, current information about predictors of mortality among these patients is scarce, and mainly derives from studies which did not use a standard definition for MDR or focused on the individual microorganisms [42].

A group of international experts came together through a joint initiative by the European and US Centers for Disease Control and Prevention in 2012. An interim standard definition of MDR proposed by the meeting was acquired non-susceptibility to at least one agent in three or more antimicrobial categories [43]. Reference 43 illustrated in detail the interim standard definition of MDR of major gram-negative bacteria, including Enterobacteriaceae, *P.aeruginosa*, and *A.baumannii. P.cepacia* and *O.anthropi* were defined as resistant to at least three of the antibiotics as MDR *P.aeruginosa* and *A.baumannii,* respectively. *S.maltophilia* was defined MDR as resistant to at least three of the antibiotics (other than antipseudomonal carbapenems) as MDR *P.aeruginosa*. After the use of an interim standard definition for MDR [44], no study has specifically been designed to identify the predictors of mortality among ASOT recipients with all types of MDR GNB so far. The purpose of this study was to describe the epidemiology, microbiology, and clinical outcomes of MDR GNB and determine the predictors of MDR GNB-related 30-day mortality in ASOT recipients.

## Methods

### Study population

This study was conducted at the Third Xiangya Hospital (Changsha) of Central South University, and Zhongnan Hospital (Wuhan) of Wuhan University, two tertiary university referral hospitals in China. From January 1, 2003 to February 29, 2016, all episodes of MDR GNB occurring in hospitalized ASOT recipients were included. Recipients who were lost to follow-up or moved out of the both hospitals within 30 days after MDR GNB or who were recipients of multiorgan transplants were excluded from the study. For the purposes of this study, recipients were divided into two groups according to their final outcomes: survival and death within 30 days after the first episode of MDR GNB. The two groups were compared to identify risk factors influencing mortality.

No liver recipients received induction therapy. The lymphocyte-depleting induction therapy was selectively used in some kidney recipients at high risk for acute rejection. All recipients enrolled were administered maintenance immunosuppressive treatment consisting of one to three drugs such as corticosteroids, a calcineurin inhibitor (e.g., tacrolimus or cyclosporine), and antimetabolic agents (e.g., mycophenolate mofetil, mizoribine, or azathioprine). Second- or third-generation cephalosporins, semisynthetic penicillins/beta-lactamase inhibitors, or carbapenems, according to the pretransplant results of cultures, were prescribed 1 h before liver transplantation and additional dose 72 h posttransplantation. Second-generation cephalosporins were prescribed 1 h before the operation and additional dose 24 h posttransplantation for kidney transplantation.

### Study design and data collection

We conducted a retrospective study of ASOT recipients who presented with MDR GNB to determine the epidemiology of pathogens and risk factors for MDR GNB-related 30-day mortality. The analysis of prognostic factors included only the first episode of MDR GNB from each patient that occurred during the observational period.

The following baseline clinical, epidemiologic and laboratory data were collected from patients’ medical records: sex; age; type and date of transplantation; type and date of diagnosis of MDR GNB; Non-fermentative bacteremia or not; ESBL(+) rods or not; carbapenem-resistant rods or not; results of antibiotic susceptibility tests; site of primary infection; presence of other concomitant BSI; empirical antimicrobial therapy; use of central venous catheters; septic shock or death information. The laboratory data were obtained within the first 24 h after the blood culture was drawn, consisting of serum creatinine and albumin levels, white blood cell, platelet and lymphocyte count. We also collected serum creatinine level at the time point of 1 week after MDR GNB. All patients were followed up for 30 days from the first episode of MDR GNB.

### Microbiologic methods

A 10-mL blood sample was injected into each bottle of a set of aerobic and anaerobic blood culture bottles and immediately transported to the clinical microbiology laboratory. Blood samples were loaded into the BACTEC 9120 blood culture system (Becton Dickinson, Cockeysville, MD, USA). Bacterial identification was performed by using the Vitek-2 system (bioMérieux, Craponne, France). In vitro antimicrobial susceptibility tests of blood isolates were performed by the Kerby-Bauer disc diffusion method and the minimum inhibitory concentration (MIC) was measured by agar dilution. The production of ESBL was detected by the confirmatory double-disk synergy test [45]. The interpretation of antimicrobial susceptibility test results followed recommendations of the National Committee for Clinical Laboratory Standards [46], which was suitable for 2003 and the Clinical and Laboratory Standards Institute [47], which was suitable for 2004–2016. Gram-negative bacteria with intermediate susceptibility to the antibiotics was considered as resistance.

### Definitions

The presence of BSI was defined according to the criteria proposed by the Centers for Disease Control and Prevention [48]. Appropriate empirical antimicrobial therapy was defined as administration of antibiotics to which the pathogen was susceptible in vitro within 48 h after the blood culture was obtained [49]. BSI was considered as nosocomially acquired if positive blood cultures were obtained in patients who had been hospitalized for 48 h or longer. Community-acquired BSI was defined if positive blood cultures were obtained within the first 48 h after hospital admission [18]. Bacteremia was defined to be early-onset occurring within the first 2 months (60 days) or less of ASOT and late-onset infection occurred beyond this time point [13]. When a positive bacterium or fungus blood culture occurred in the setting of documented MDR GNB, the modifier “presence of other concomitant BSI” was applied to that infection.

Septic shock was diagnosed in recipients who had a positive blood culture and occurred persistent dysfunction of at least one organ owing to hypoperfusion unresponsive to intravenous fluid challenge [50]. MDR GNB-related mortality was regarded if death was correlated with clinical signs of MDR GNB without evidence of any other cause [51].

### Statistical analysis

Statistical analyses were executed with the statistical package SPSS for Windows, version 22.0 (IBM SPSS Statistics, IBM Corporation, Armonk, NY, United States). Continuous variables were listed as means ± standard deviations or medians (1st-3rd quartile) according to the distribution. Category variables were compared by Pearson’s *χ* ^2^ test or Fisher’s exact test when appropriate. To determine the association between demo/clinical variables and MDR GNB-related 30-day mortality, all covariates associated with a *P* value of <0.05 in the univariate analysis were selected for multivariate logistic regression analysis. The final model was obtained with a forward stepwise regression approach. Odds ratio (OR) with 95% confidence intervals (CI) for the effect of demographic, clinical and laboratory predictors on mortality were evaluated using logistic regression. The method of multivariable multicollinearity diagnostics was used to detect if any of the risk factors for bacteremia-associtated mortality including presence of other concomitant BSIs, nosocomial infection, creatinine > 1.5 mg/dL and septic shock was an independent risk factor. All* P* values were bicaudate and a value of *P* < 0.05 was considered to be statistically significant.

## Results

During the 13-year study period, we performed 1681 ASOT in the Third Xiangya Hospital, including 1465 kidney, 210 liver, five simultaneous liver-kidney, and one simultaneous kidney-pancreas transplantations, and 488 ASOT in Zhongnan Hospital, consisting of 346 kidney, 140 liver, and two simultaneous liver-kidney transplantations. A total of 327 episodes of BSI including bacteremia and fungemia occurred in 227 (10.5%) of 2169 ASOT recipients.

A total of 168 episodes of GNB were diagnosed among these patients. Ninety-nine MDR gram-negative isolates were isolated from 91 (4.6%) ASOT recipients with BSI. The median age of these 91 recipients was 45 years (interquartile range 35–54) with a male predominance (68.1%). A significantly higher proportion of liver recipients (44 [12.3%] of 357 patients) were affected by MDR GNB when compared with renal patients (47 [2.6%] of 1812 subjects) (*P* < 0.001). The median time after the transplantation for the diagnosis of MDR GNB was 26 days (interquartile range: 7–81 days). The median time for the diagnosis of the first episode of MDR GNB was significantly longer among renal recipients (69 days; interquartile range: 27–184 days) than in liver recipients (11 days; interquartile range: 3–26 days) (*P* < 0.001). Of these 91 ASOT recipients, 74 were classified as nosocomially acquired MDR GNB with a mortality of 47.3% (35/74) which was higher than the mortality of 5.9% (1/17) caused by community-acquired MDR GNB. Appropriate empiric antibiotic therapy was prescribed in only 57 (62.6%) patients.

Seventy-eight cases (85.7%) had a fever at the onset of MDR GNB. The predominant primary sites of infection in the first episode of MDR GNB were lungs (40.7%) and intra-abdomen/biliary tract (23.1%). Five (5.5%) primary sites of infection were vascular catheters. Eighty-three patients experienced a single episode of MDR GNB and eight cases experienced two episodes. Thirty-eight (41.8%), 73(80.2%) and 36 (39.6%) patients infected by carbapenem-resistant rods, ESBL(+) rods and non-fermenters, respectively. Thirty-five cases (38.5%) experienced MDR GNB presenting with other concomitant BSI. There were 33 (36.3%) septic shocks at the onset of MDR GNB. MDR GNB-related 30-day mortality after the first episode of MDR GNB was 39.6% (36 deaths). The demographic, clinical, and laboratory characteristics of the patients are presented in Table 1.

**Table 1:** Demographic, laboratory and clinical variables of 91 ASOT recipients with MDR GNB

Characteristics	Value
Age, median years (IQR)	45 (35-54)
Sex, number of male (%)	62 (68.1)
Temperature of 40 °C or greater, no. of cases (%)	12 (13.2)
Nosocomial origin, no. of cases (%)	74 (81.3
Inappropriate antimicrobial use, no. of cases (%)	34 (37.4)
Septic shock, no. of cases (%)	33 (36.3)
The type of donor, no. of cases (%)	
Living-related	11 (12.1)
DCD	45 (49.5)
Deceased	35 (38.5)
The type of transplantation, no. of cases (%)	
Liver	44 (48.4)
Kidney	47 (51.6)
Site of primary infection, no. of cases (%)	
Lung	37 (40.7)
Intra-abdominal/biliary	21 (23.1)
Urinary tract	13 (14.3)
Vascular catheter	5 (5.5)
Gastrointestinal tract	3 (3.3)
Unknown	13 (14.3)
Type of organisms, no. of cases (%)	
Monomicrobial	83 (91.2)
Polymicrobial	8 (9.8)
Non-fermentative bacteremia, no. of cases (%)	
Yes	36 (39.6)
No	55 (60.4)
ESBL (+) rods, no. of cases (%)	
Yes	73 (80.2)
No	18 (19.8)
Carbapenem-resistant rods, no. of cases (%)	
Yes	38 (41.8)
No	53 (58.2)
Time of bacteremia onse, no. of cases (%)	
< 2 months posttransplant (early-onset)	62 (68.1)
≥ 2 months posttransplant (late-onset)	29 (31.9)
Laboratory variables, no. of cases (%)	
Platelet count < 50000/mm^3^	39 (42.9)
Lymphocyte count < 300/mm^3^	32 (35.2)
Albumin < 30 g/L	16 (17.6)
WBC count > 15000/mm^3^	27 (29.7)
Creatinine > 1.5 mg/dL at onset of bacteremia	42 (46.2)
Creatinine at 1 week after bacteremia	
> 1.5 mg/dL	26 (28.6)
≤ 1.5 mg/dL	49 (53.8)
Missing due to death within 1 week after bacteremia	16 (17.6)
Related mortality, no. of cases (%)	36 (39.6)

*ASOT* abdominal solid organ transplantation, *DCD* donation after cardiac death, *ESBL* extended-spectrum beta-lactamase, *MDR* multidrug-resistant, *GNB* gram-negative bacteremia, *IQR* interquartile range, *WBC* white blood cells

Ninety-nine MDR gram-negative bacteria were isolated from these 91 ASOT recipients with BSI, with a predominance of *E.coli* (29 isolates, 29.3%), *A.baumanii* (24 isolates, 24.2%), *E.cloacae* (11 isolates, 11.1%), and *K.pneumoniae* (ten isolates, 10.1%). Table 2 listed in detail the classification and percentage of 99 MDR gram-negative bacteria isolated.

**Table 2:** Classification and percentage of 99 multidrug-resistant gram-negative bacteria isolated from 91 recipients with bacteremias

Gram negative bacilli	Strain (*n* = 99)	Percentage (%)
*E.coli*	29	29.3
*A.baumanii*	24	24.2
*E.cloacae*	11	11.1
*K.* *pneumoniae*	10	10.1
*S.maltophilia*	7	7.1
*P. aeruginosa*	6	6.1
*O.anthropi*	4	4.0
*K.ozaenae*	2	2.0
*C. farmeri*	1	1.0
*E.aerogenes*	1	1.1
*P.cepacia*	1	1.1
*S.marcescens*	1	1.0
*S.rubidaea*	1	1.0
*P.mirabilis*	1	1.0

The association of the studied covariates with mortality after the first episode of MDR GNB by using the univariate and multivariate analyses is shown in Table 3. The univariate analysis identified the variables as predictors of MDR GNB-related mortality included lung focus (*P* = 0.001),nosocomial origin (*P* = 0.002), graft from donation after cardiac death or deceased donors (*P* = 0.023), presence of other concomitant BSI (*P* < 0.001), temperature of 40 C or greater at the onset of BSI (*P* = 0.039), creatinine > 1.5 mg/dl at onset of MDR GNB (*P* = 0.006), albumin < 30 g/L (*P* = 0.009), platelet count < 50,000/mm^3^ (*P* < 0.001), and septic shock (*P* < 0.001). A factor that tended to be associated with higher mortality was lymphocyte count < 300/mm^3^ (*P* = 0.051). Septic shock (OR = 160.463, 95% CI = 19.377–1328.832, *P* < 0.001), as well as creatinine > 1.5 mg/dl at onset of bacteremia (OR = 24.498, 95% CI = 3.449–173.998, *P* = 0.001), nosocomial origin (OR = 23.963, 95% CI = 1.285–446.991, *P* = 0.033), and presence of other concomitant BSI (OR = 27.074, 95% CI = 3.937–186.210, *P* = 0.001) remained statistical significance in the multivariate logistic regression analysis. The method of multivariable multicollinearity diagnostics was used to detect if any of the risk factors for bacteremia-associtated mortality including presence of other concomitant BSIs, nosocomial infection, creatinine > 1.5 mg/dL and septic shock was an independent risk factor. We found that all variance inflation factors wrer less than 1.2, all condition indexs were less than seven and none of eigenvalue was equal to 0, which indicated that these risk factors were independent.

**Table 3:** Risk factors for bacteremia-related mortality in ASOT recipients with MDR GNB

Characteristics	Related mortality	Survival	*P*	OR (95% CI)
Total, n (%)	36 (39.6)	55 (60.4)		
Univariate analysis				
Age ≥ 40 year	25 (69.4)	35 (63.6)	0.568	
Male sex	24 (66.7)	38 (69.1)	0.808	
Temperature ≥ 40 °C	8 (22.2)	4 (7.3)	0.039	
Inappropriate empirical antibiotics	14 (38.9)	20 (36.4)	0.808	
Nosocomial infection	35 (97.2)	39 (70.9)	0.002	
Graft from DCD or deceased donors	17 (47.2)	39 (70.9)	0.023	
Liver transplant	20 (55.6)	24 (43.6)	0.266	
Lung focus	22 (61.1)	15 (27.3)	0.001	
Presence of other concomitant BSIs	22 (61.1)	13 (23.6)	<0.001	
Non-fermentative bacteremia	18 (50.0)	18 (32.7)	0.099	
ESBL (+) bacteremia	31 (86.1)	42 (76.4)	0.254	
Carbapenem-resistant bacteremia	16 (44.4)	22 (40.0)	0.674	
Late-onset infection	12 (33.3)	17 (30.9)	0.808	
Septic shock	28 (77.8)	5 (9.1)	<0.001	
Platelet count < 50,000/mm^3^	26 (72.2)	13 (23.6)	<0.001	
Lymphocyte count < 300/mm^3^	17 (47.2)	15 (27.3)	0.051	
Albumin < 30 g/L	11 (30.6)	5 (9.1)	0.009	
WBC count > 15,000/mm^3^	12 (33.3)	15 (27.3)	0.277	
Creatinine > 1.5 mg/dL	23 (63.9)	19 (34.5)	0.006	
Multivariate analysis				
Presence of other concomitant BSIs			0.001	27.074 (3.937–186.210)
Nosocomial infection			0.033	23.963 (1.285–446.991)
Creatinine > 1.5 mg/dL			0.001	24.498 (3.449–173.998)
Septic shock			<0.001	160.463 (19.377–1328.832)

*ASOT* abdominal solid organ transplant, *DCD* donation after cardiac death, *ESBL* extended-spectrum beta-lactamase, *MDR* multidrug-resistant, *GNB* gram-negative bacteria, *OR* odds ratio, *CI* confidence interval, *BSIs* bloodstream infections, *WBC* white blood cells

## Discussion

MDR gram-negative isolates, including carbapenem-resistant *K.pneumoniae* and ESBL- or carbapenemase- producing Enterobacteriaceae, are an emergent problem, with limited therapeutic options. It maybe prolong the hospital stay, increase the difficulty of treatment and the risk of death. SOT recipients are especially at high risk for infection by MDR gram-negative bacteria due to numerous hospitalizations, surgical interventions, preexisting and posttransplant immunosuppression, the use of invasive devices and frequent antibiotic exposures.

Understanding the epidemiology and the risk factors of mortality associated with MDR GNB may facilitate guiding appropriate initiation of antibiotic therapy, and reducing the risk factors associated with mortality [44]. Here we specially described a large retrospective cohort of ASOT recipients and their incidence of all MDR GNB over a 13-year period. *E.coli* and *A.baumanii* were the predominant MDR gram-negative bacteria among these ASOT population with BSI. Our data confirmed the profound impact of MDR GNB on mortality. The incidence of MDR GNB was higher in liver recipients (12.3%) than in renal recipients (2.6%). More severe disease and exposure to multiple antimicrobial therapies before and after liver transplantation, technical complexity of liver transplant surgery, prolonged operative time, more transfusion during surgery, longer postoperative intensive care unit stay and admission days, and invasive diagnostic procedures after liver transplantation may account for this difference.

To our best knowledge, this is the first study to specifically address the risk factors for attributable mortality among ASOT recipients with all types of MDR GNB, after an interim standard definition of MDR was proposed in 2012 [43]. Here, we observed an occurrence rate of 4.6% and a mortality rate of 39.6% among ASOT patients affected by MDR GNB, a finding consistent with previous reports. This current analysis, however, includes a larger number of ASOT recipients with all types of MDR GNB than previous studies, which generally addressed only one type of MDR gram-negative bacteria such as carbapenem-resistant *K.pneumoniae* or ESBL-producing Enterobacteriaceae or all kinds of infections due to MDR gram-negative isolates [35–40, 52].

We found, in the current study, that nosocomial origin, as well as the presence of other concomitant bloodstream infections, increased creatinine level and septic shock, all of which indicated a severity of illness, more hospitalized or invasive procedures and particularly difficult to treat, were the main predictors of MDR GNB-related 30-day mortality. We found that septic shock led to a 160-fold increase in mortality and was the strongest predictor of patient outcomes in multivariate models. This association of septic shock with mortality echoed the findings of our previous study [53–55] and other studies [26, 42, 56, 57] based on SOT recipients with BSI. Septic shock is the most severe complication in the context of BSI in SOT recipients and improving the management of septic shock remains a great challenge [2, 42]. It is not surprising because septic shock is a manifestation of the severity of illness. Aguiar EB et al. also reported factors associated with clinical severity including the Pitt bacteremia score and being on mechanical ventilation at the time of infection diagnosis were the main predictors of mortality in SOT recipients with bacteremia caused by ESBL-producing Enterobacteriaceae [35]. This finding can alert clinicians to the importance of early therapy [58], including protocolized resuscitation with intravenous fluids and early empirical antibiotic therapy, which may reduce the mortality associated with severe sepsis and septic shock, which occur in nearly 15% of bacteremic infections in SOT recipients and have a mortality rate of 50% [7].

In our present study, nosocomially acquired MDR GNB had a higher mortality of 47.3% than the mortality of 5.9% caused by community-acquired MDR GNB, which was consistent with a recent report from Thailand in the general population suggesting that the mortality in MDR hospital-acquired bacteremia was higher than MDR community-acquired bacteremia (53% vs. 35%) [59]. Thus, nosocomial infection was also associated with MDR GNB-related mortality in the present study, which was in line with a USA study of SOT recipients with BSI conducted by Hsu J et al. [21]. MDR gram-negative bacilli have emerged as a significant pathogen responsible for nosocomially acquired infections [60]. It is not surprising that nosocomial infection predicted mortality given that it indicated more exposure to multiple antimicrobial therapies and particularly difficult to treat. Other previous studies also claimed that when compared to other BSI in SOT patients, nosocomial BSI was associated with an increased risk of septic shock and failure of clinical cure [2, 7, 61, 62]. Given nosocomial MDR GNB can be limited by specific preventive measures, we provided fertile ground that MDR GNB-related morbidity and mortality after ASOT could be further decreased [63].

We also revealed that increased serum creatinine level affected survival of ASOT recipients with MDR GNB. This finding was accordant with previous studies of SOT recipients with BSI revealing that renal dysfunction was related to increased mortality [5, 33, 53, 64]. A recent study of SOT recipients with BSI also reported that renal impairment (a serum creatinine level of >1.5 mg/dL) was more frequent among non-survivors [42]. Renal insufficiency was also reported as a predictor of mortality among hematopoietic stem cell transplant patients with invasive *Aspergillosis* infections [65–67]. There are multiple etiologies for renal impairment in transplant recipients, including veno-occlusive disease, drug toxicities, and sepsis, all of which are associated with increased mortality [66].

Consistent with a previous study [5] based on SOT recipients with BSI, the present study indicated that presence of other concomitant BSI was another risk factor which contributed to mortality. Our previous studies found polymicrobial was a risk factor for BSI-related mortality in deceased donor liver or SOT recipients in the univariate analysis whereas this association did not remain in the multivariate analysis [51, 53].

### Limitation

Our study has several limitations that should be mentioned. First, this study was limited by its retrospective study nature owing to insufficient information, inaccurate written records, or selection biases. In order to avoid these limitations, prospective studies are needed to validate the risk factors for mortality proposed by the current study in future research. Second, this study is also limited due to the relatively small sample size of cases and deaths. Therefore, the power of mortality analyses is limited, as indicated by the wide range of some of the associated confidence intervals. This is a common problem in studies evaluating risk factors for mortality due to MDR organisms. Further well-designed studies with larger sample size were warranted, especially studies exploring the association of demographic, clinical and laboratory variables with additional outcomes caused by MDR GNB. Third, we did not have information of patient colonization before transplantation. Fourth, it will be helpful to include more data of different time points of the creatinine level because, with time going on, the increased level may be varying. We intended to include creatinine > 1.5 mg/dl at 1 week after bacteremia in the univariate analysis. However, Six recipients died in the first day, three the second day, four the third day, two the fourth day, and one the fifth day after MDR GNB, which produced 16 missing values thus we eventually did not include this variable in the univariate analysis. Finally, we analyzed a heterogeneous group of ASOT recipients who may have had their own specific risk factors for mortality. Although due to the retrospective data collection, all reported findings should be interpreted with care, a double-center setting in different cities in China and a long 13-year period of study can benefit its generalization of the study findings. Further strategies to reduce the incidence of posttransplant BSI in ASOT recipients and to control the spread of MDR gram-negative bacteria, such as the administration of a short duration of antibiotic prophylaxis, a timely removal of unnecessary catheters, and avoiding the overuse of antibiotics and antifungal drugs are needed to reduce the impact of MDR GNB on allograft and patient outcomes [68].

## Conclusion

MDR GNB was associated with high morbidity and mortality in ASOT recipients, with a predominant causative organisms being *E.coli* and *A.baumanii*. Nosocomial origin, presence of other concomitant BSI, along with increased creatinine level and septic shock after ASOT were the risk factors for MDR GNB-related mortality in ASOT recipients. Recognition of these factors is useful in identifying individuals who are at risks of mortality.

## Abbreviations

ASOT: Abdominal solid organ transplantation
BSI: Bloodstream infection
CI: Confidence intervals
ESBL: Extended-spectrum β-lactamases
GNB: Gram-negative bacteremia
MDR: Multidrug-resistant
MIC: Minimum inhibitory concentration
OR: Odds ratio
SOT: Solid organ transplantation
